# Comparison Between Manual and (Semi-)Automated Analyses of Esophageal Diaphragm Electromyography During Endurance Cycling in Patients With COPD

**DOI:** 10.3389/fphys.2019.00885

**Published:** 2019-07-10

**Authors:** Sauwaluk Dacha, Luc Janssens, Antenor Rodrigues, Zafeiris Louvaris, Lotte Janssens, Rik Gosselink, Daniel Langer

**Affiliations:** ^1^Department of Rehabilitation Sciences, Faculty of Movement and Rehabilitation Sciences, Research Group for Rehabilitation in Internal Disorders, KU Leuven, Leuven, Belgium; ^2^Respiratory Rehabilitation and Respiratory Division, University Hospital Leuven, Leuven, Belgium; ^3^Department of Physical Therapy, Faculty of Associated Medical Science, Chiang Mai University, Chiang Mai, Thailand; ^4^Faculty of Engineering Technology, Electrical Engineering (ESAT) TC, KU Leuven, Leuven, Belgium; ^5^Laboratory of Research in Respiratory Physiotherapy, Department of Physiotherapy, Universidade Estadual de Londrina, Londrina, Brazil; ^6^GP Livens and M Simou Laboratories, First Department of Critical Care Medicine and Pulmonary Services, Athens University Medical School, Evangelismos Hospital, Athens, Greece; ^7^REVAL Rehabilitation Research Center, Faculty of Rehabilitation Sciences, Hasselt University, Hasselt, Belgium

**Keywords:** electromyography, electrocardiography, diaphragm electromyography, chronic obstructive pulmonary disease, respiratory muscle training

## Abstract

**Background:** Electrocardiogram (ECG) contamination is present in diaphragm electromyography (EMGdi) recordings. Obtaining EMGdi without ECG contamination is crucial for EMG amplitude analysis. Manually selecting EMGdi in between QRS complexes has been most commonly applied in recent years (manual method). We developed a semi-automated analysis method based on Least Mean Square Adaptive Filtering combined with a synchronously recorded separate ECG channel to remove ECG artifacts from the EMGdi signals. We hypothesized that this approach would shorten analysis duration and might minimize the potential for inter-rater disagreement.

**Aims:** We aimed to evaluate agreement between the semi-automated method and the manual method and inter-rater reliability of the manual method.

**Methods:** Electromyography signals of seven patients with COPD were recorded using an esophageal catheter during an exercise test on a cycle ergometer. Four patients subsequently participated in an inspiratory muscle training (IMT) program for 8 weeks. After IMT, the tests were repeated. EMGdi/EMGdiMax as obtained either manually by the two assessors or retrieved from the semi-automated method were compared.

**Results:** Semi-automated EMGdi/EMGdiMax agreed well with values obtained by one of the two manual assessors (assessor 1) both at pre-intervention measurements (mean difference −0.5%, 95% CI: −19.6 to 18.6%) and for the pre/post IMT differences (mean difference 1.2%, 95% CI: −16.8 to 19.2%). Intra-class correlation coefficients between methods were 0.96 (95% CI: 0.94–0.97) at pre-intervention measurements and 0.78 (95% CI: 0.58–0.89) for pre/post IMT differences (both *p* < 0.001). EMGdi/EMGdiMax from assessor 2 was systematically lower than from assessor 1 and agreed less well with the semi-automated method both at pre-intervention measurements (mean difference: 9.3%, 95% CI: −11.4 to 29.9%) and for pre/post IMT differences (mean difference 7.0%, 95% CI: −20.4 to 34.4%). Analysis duration of the semi-automated method was significantly shorter (29 ± 9 min) than the manual method (82 ± 20 min, *p* < 0.001).

**Conclusion:** The developed semi-automated method is more time efficient and will be less prone to inter-rater variability that was observed when applying the manual analysis method. It is, therefore, proposed as a new standard for objective EMGdi amplitude analyses in future studies.

## Introduction

Electromyography (EMG) is an assessment of muscle activation by recording the electrical activity of the muscle tissue. Assessments of diaphragm EMG (EMGdi) amplitude are frequently applied in both clinical and research settings, where they can serve as an indirect measure of neural respiratory drive (NRD) during different conditions such as resting breathing, exercise breathing, or during sleep (Luo and Moxham, 2005; Luo et al., 2011; Xiao et al., 2015; Steier et al., 2017). EMGdi can be recorded either via surface electrodes placed on the chest wall, with needle electrodes inserted into the costal diaphragm, or with an esophageal catheter equipped with EMG electrodes (Sinderby et al., 1998; Duiverman et al., 2004; Luo et al., 2008). The EMGdi recording contains artifacts from the power line, from movement, and from cardiac activity. Movement artifacts are associated with very low frequencies and can be easily removed by applying high pass filtering at 20 Hz. However, the cardiac activity artifacts, as detected by electrocardiogram (ECG), is more difficult to remove because of the overlapping bandwidth spectrum between ECG and EMGdi. The majority of the EMGdi signal is concentrated in the bandwidth between 20 and 250 Hz, while the bandwidth of the ECG frequency spectrum lies between 0 and 100 Hz (Schweitzer et al., 1979). It is crucial to obtain the EMGdi signal without the ECG contamination, to ensure the accuracy of the EMGdi signal (Levine et al., 1986; Zhou and Kuiken, 2006; Luo et al., 2008). Separating ECG from EMGdi is particularly challenging in EMG amplitude analyses, especially during exercise, since the EMGdi amplitude can be larger than the ECG. This makes it more difficult to identify ECG artifacts within the EMG signal.

One widely used method to obtain the EMGdi signal without ECG contamination is to manually select EMGdi data in between QRS complexes (Luo and Moxham, 2005; Luo et al., 2008; Jolley et al., 2009; Reilly et al., 2013; Schaeffer et al., 2014; Langer et al., 2018). By placing a separated time-synchronized ECG channel next to the EMG channel, the ECG channel is visually identifiable, thereby allowing to retrieve the EMGdi in between QRS complexes. However, there are some limitations to this method. First, this method is time-consuming, especially for recordings that contain many breathing cycles such as during exercise. Second, based on the experience in our research group, it might be subjective to inter-rater variability since the retrieved data can vary depending on the judgment of the assessor. Inter-rater variability could arise from the fact that several EMGdi parts are available to choose from in between QRS complexes during every inspiration. No specific instructions are currently available as to which interval should be preferably selected under these circumstances while selection of either ascending, descending, or peak intervals of the uncontaminated signal might result in vast differences in the recorded EMG amplitude. Selection width of the chosen interval while avoiding artifact to either side of the selected interval might be another factor that could explains the inter-rater variability of the obtained EMG amplitude for a given breath. A final limitation of the manual method is that EMGdi activity “buried” in the ECG signal cannot be selected. Depending on the location of the QRS complexes the data outside of contaminated area might not be the best representation of the actual EMG amplitude (e.g., the part containing the highest amplitude of the signal might not be available to select). This might be especially problematic during exercise, when several heartbeats typically occur during a single inspiration.

Several methods have been previously applied to automatically deduct or remove ECG artifacts from EMGdi signals. However, the majority of methods does not rely on ECG data from a separately collected ECG channel. These methods typically suffer from problems with frequency-overlapping, difficulties in waveform identification, and processing difficulties due to the sometimes smaller amplitude of the ECG signal in comparison to the EMG signal (e.g., during near maximal diaphragm activation throughout exercise hyperpnea) (Schweitzer et al., 1979; Zhou and Kuiken, 2006; Mak et al., 2010; Willigenburg et al., 2012). Bloch suggested using a separate and simultaneous recording of a time-synchronized ECG channel to avoid these problems (Bloch, 1983). For analysis, he proposed to initially use the amplitude threshold to identify the QRS complex of the ECG, followed by applying a least squares subtraction on the time domain to remove ECG artifacts (Bloch, 1983). This method introduced by Bloch has not been extensively evaluated or validated especially not for EMGdi recordings of resting and exercise breathing obtained with an esophageal catheter.

Up to now, there is no gold standard method available for removing ECG artifacts while analyzing EMGdi amplitude data. From the reviewed methods above, manually selecting EMGdi in between QRS complexes has so far been the most applied method. This method will be mentioned onward as the “manual” method. Because of the shortcomings of the manual method, we were interested in developing and evaluating an alternative method that could potentially shorten the duration of the analysis and overcome several problems related to the expected inter-rater ambiguity that seems inherent to the somewhat subjective judgments that have to be made while applying the manual analysis method. Therefore, we developed a custom “semi-automated method” based on a Least Mean Square (LMS) Adaptive Filtering method (Bloch, 1983) combined with a synchronously recorded, separated ECG channel. We aimed to compare this “semi-automated method” with results obtained from the manual method. In addition, we also aimed to formally study the degree of inter-rater variability that can be expected when applying the manual analysis method. Responsiveness (i.e., the ability of a measure to detect change) is an important feature of assessment methods that needs to be evaluated separately from reliability and validity (Husted et al., 2000).The degree of agreement between methods was therefore evaluated both cross-sectionally (i.e., of data obtained at a single point in time) to evaluate validity and reliability as well as by comparing changes in activation observed after an intervention period between methods to evaluate and compare responsiveness.

Accordingly, the aims of this study were the following: (1) to investigate the inter-rater reliability of the manual method of EMGdi amplitude analysis and (2) to explore the agreement between the manual and the proposed semi-automated analysis method of EMGdi amplitude signals both cross-sectionally (to evaluate validity) and of changes in response to an intervention (to evaluate responsiveness).

## Materials and Methods

### Study Design and Subjects

Clinically stable patients with moderate to severe COPD were included in this study. Data were retrieved from patients who had been enrolled in a clinical study (ClinicalTrials.gov Identifier: NCT03240640). The Ethical Committee Research of KU Leuven/UZ Leuven, Belgium approved the study (S58513). All participants signed written informed consent. EMGdi was recorded during a constant work rate cycle ergometer (CWR) test before and after 8 weeks of inspiratory muscle training (IMT). The EMGdi data were first analyzed using the manual method by two independent assessors. The same data were then analyzed again using the semi-automated method. Comparisons were made both between the results obtained by the two assessors using the manual analyzing method as well as between results obtained by both manual assessors and from the semi-automated method. The details of each analysis method are described below. Interim analysis of these data has been presented at ERS International Congress 2018 (Dacha et al., 2018).

### Pulmonary Function and Respiratory Muscle Function Measurements

Pulmonary function testing (MasterScreen Body, CareFusion, Höchberg, Germany) was performed according to ERS guidelines (Miller et al., 2005; Wanger et al., 2005; Graham et al., 2017). Maximal inspiratory, expiratory mouth pressures (MIP and MEP) and transdiaphragmatic pressure (PdiMax) during sniff maneuver were assessed according to international guidelines (American Thoracic Society/European Respiratory Society [ATS/ERS], 2002).

### EMG Recording and Analysis

#### Esophageal Catheter and Positioning

**FIGURE 1:**
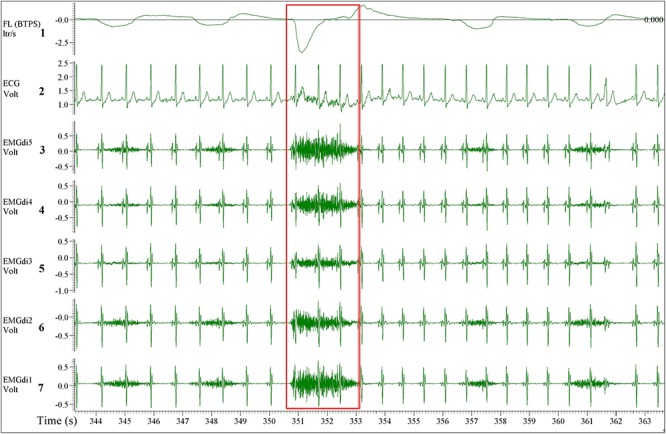
A five diaphragm EMG (EMGdi) channel recording contains electrocardiogram (ECG) artifacts during resting breathing. From top to bottom the channels are as follow; Channel 1: respiratory flow (l/s; negative flow indicating the inspiratory cycle), Channel 2: ECG recording (volt), Channels 3–7: EMGdi recordings. The correct positioning of the catheter is shown when the largest EMGdi amplitudes are in the outer EMGdi channels (3 and 7), and the smallest amplitude is in the middle channel (5). The inspiratory capacity (IC) maneuver is highlighted in the red box indicated by the higher flow, which accompanies the maximal activation of the diaphragm (EMGdiMax).

A multipair-esophageal electrode catheter (Yinghui Medical Equipment Technology Co., Ltd., Guangzhou, China) was used to assess EMGdi. The catheter is approximately 60 centimeters long, two millimeters in diameter, and is equipped with five EMG electrode pairs feeding five EMGdi channels. The catheter was inserted nasally and then swallowed by the patient. The positioning of the catheter was performed according to procedures established in previous studies (Luo and Moxham, 2005; Luo et al., 2011). In short, the patient was asked to perform several slow maximal inspiratory capacity (IC) maneuvers (an inspiration through the open mouth from the functional residual capacity to total lung capacity). The best position was determined as the location which the largest EMGdi amplitudes were recorded from the outer electrode pairs and the smallest from the middle pairs (Figure 1; Luo et al., 2001, 2011; Luo and Moxham, 2005). After positioning, the catheter was secured by taping one end onto the patient’s nose.

#### EMGdi Sampling and Processing

The EMGdi signals were first amplified (Biomedical amplifier, Guangzhou, China), sampled at 2000 Hz by a data acquisition system (Micro1401-3, Cambridge Electronic Design Limited, Cambridge, United Kingdom) and then processed with a specific software package (Spike 2, Cambridge Electronic Design Limited, Cambridge, United Kingdom). During processing the raw EMGdi data were first high pass filtered at 20 Hz to minimize motion artifacts and then transformed into “Root Mean Square” (RMS). The EMGdi signals recorded during breathing were then normalized by presenting the recorded value relative to the signal obtained during maximal activation; EMGdi/EMGdiMax%. The highest EMGdi signal obtained from any of the five channels during each subsequent breath was retrieved for analyses. Maximal activation of the diaphragm was obtained during typical (i.e., fast) exercise IC maneuvers, either during resting breathing or during exercise breathing (Sinderby et al., 1998). The patients were asked to perform these IC maneuvers every minute during the three resting minutes preceding the cycling test, and every other minute during the cycling test. The largest RMS amplitude obtained during any of the recorded IC maneuvers was selected as EMGdiMax.

#### ECG Removal With the Manual Method

**FIGURE 2:**
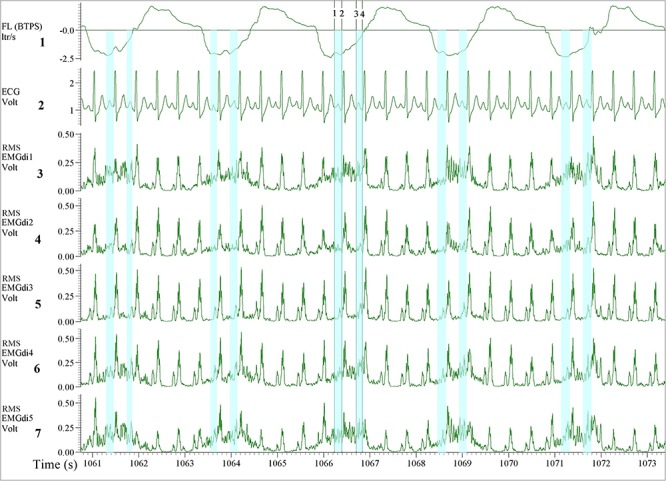
Illustration of the diaphragm EMG (EMGdi) recordings toward the end of the (symptom limited) cycling test. Channel 1: respiratory flow (l/s; negative flow indicating the inspiratory cycle), Channel 2: electrocardiogram (ECG) recording (volt), Channels 3–7: RMS EMGdi recordings. For the data analysis using the manual method, the mean values of EMGdi in between QRS complexes during the inspiratory cycle were selected. The periods highlighted in light blue are the possible periods that could be chosen without ECG contamination in each inspiratory cycle. The average of EMGdi of five consecutive breaths was used as a representative value of EMGdi of that minute. Vertical cursors 1, 2, 3, and 4 were used as a tool during manual analysis to retrieve mean values of EMGdi in the selected period.

From the processed data, both assessors were instructed to perform the manual analysis of the EMGdi signals in agreement with previously published methods. They were instructed to extract the EMGdi signals from segments of inspiratory cycles between QRS complexes (Luo et al., 2001, 2011; Luo and Moxham, 2005; Reilly et al., 2013); however, reflecting previously published methods, no instructions were given with regards to handling possible residual interference by P, T, or U waves (Figure 2). Thus, we cannot exclude the possibility of such interference within the manually derived EMGdi signal. Noteworthy, the values that have been extracted between QRS complexes in most previous literature is the peak RMS EMGdi signal of a given breath (Sinderby et al., 1996; Luo and Moxham, 2005; Reilly et al., 2011, 2012, 2013). However, as we were interested in measuring an estimate of the integral (i.e., mean) respiratory neural drive of the inspiratory cycle of a given breath, the mean value between QRS complexes that would represent the integral activation was used for analysis instead of the peak value (Langer et al., 2018). The time-synchronized flow and ECG channels were used as a guide for EMGdi selection. Five representative (preferably consecutive) breaths toward the end of each minute were selected. The choice of using five breaths toward the end of a given minute is based on in-house previous analysis that shows the mean value obtained from the last five breaths of a given minute being similar to the average of the values obtained from the last 30 s of the same minute. Breaths were disregarded in case they represented short sighs or included visible noise (e.g., from coughing) or if they were visibly different compared to surrounding breaths. The average of EMGdi of these five breaths was used as representative of diaphragm activation of each minute of the cycling test.

#### ECG Removal With the Semi-Automated Method

To perform semi-automated ECG exclusion using the newly developed algorithm several steps had to be executed. First, in the data acquisition software (Spike 2, Cambridge Electronic Design Limited, Cambridge, United Kingdom), the following recording channels were selected and exported at 1000 Hz into a text file using the export option from the data acquisition software: ECG, EMGdi (five channels),respiratory flow and volume, and a channel including event markers. These markers were manually inserted during the test to spot the transition from one condition to another during the test. The entire length of the data file, including the resting period before cycling, 1 min of unloaded cycling and all minutes of loaded cycling until symptom limitation were exported. The exported file was then imported into LABVIEW (National Instruments, Austin, TX, United States) software. The waveforms of the recorded channels was also visible in LABVIEW for inspection.

To reduce the ECG content of the diaphragm EMG channels we used a method called “adapted filtering.” The LMS Adaptive Filter is a pattern recognition algorithm, which is available in the LABVIEW software. This method is a filtering method in the frequency domain that aims to remove the ECG frequency content out of the total signal. The filter was tuned to comply with the minimum error and consequently delivered the best results to remove the ECG component from the recorded signal. We used a filter length of 70 and a step size of 0.01 as the most optimal coefficients for this analysis. A separate channel was used to record the ECG synchronously to tune the coefficients of the Finite Impulse Response (FIR) filter continuously. In this way, the removal was very precise, even though the heart rhythm was changing throughout the test. More detail concerning the LMS Adaptive Filtering can be found in this link http://www.ni.com/example/31220/en/.

**FIGURE 3:**
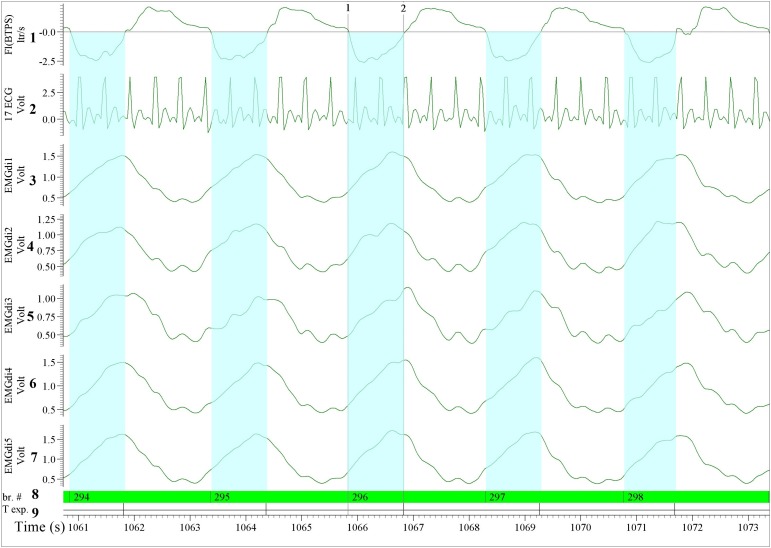
Illustration of the diaphragm EMG (EMGdi) recordings toward the end of the (symptom limited) cycling test (period comparable to Figure 2). For the data analysis using the semi-automated method. Channel 1: respiratory flow (l/s; negative flow indicating the inspiratory cycle), Channel 2: ECG recording (volt); The absolute ECG values (volt) from this re-imported data after having been processed in LABVIEW using the semi-automatic algorithm were transformed into an abstract unit. Therefore, the signal appeared to be distorted and cannot be compared directly to the pre-processed ECG signal, Channels 3–7: the processed EMGdi data from our customized algorithm without ECG contamination in the diaphragmatic EMG signal (EMGdi). Channel 8: br.#, beginning of the inspiratory cycle and Channel 9: T exp., beginning of the expiratory cycle indicates the inspiratory and expiratory cycle of each breath which was marked automatically by the program. The EMGdi during a full inspiratory cycle can be selected (highlighted in light blue). The average of the mean EMGdi from every inspiratory cycle in each minute was analyzed. Vertical cursors 1 and 2 indicate a longer period that the value of EMGdi could be retrieved compare to the same breath in Figure 2 that only shorter periods were available.

The ECG filtering algorithm was pre-set in the LABVIEW software, the ECG channel was recognized automatically by the algorithm. After importing the data, the assessor selected the ECG exclusion option on all EMGdi channels. The algorithm then automatically ran and the assessor was notified when the “cleaned” EMGdi data were ready to be retrieved. These results were then saved in a separate text file. This text file containing the cleaned EMGdi data was then re-imported into the data acquisition software (Spike 2). The assessor then used a respiratory script application (commercially available upon purchase of the software) available in the data acquisition software (spike 2) to further process the data. The respiratory script automatically marks the inspiratory and the expiratory cycle of each breath throughout the selected recording interval based on the respiratory flow signal (i.e., based on zero-flow points). The mean of the integrated EMGdi signal (RMS) during every inspiratory cycle (marked periods) throughout the cycling test was then automatically calculated and exported to an excel sheet. The values of these mean integrated EMGdi signals of every breath could not be manipulated by the assessor. The assessor then identified the resting and exercise period of each test and each minute of the test was manually marked. The average of the mean of integrated RMS from every inspiratory cycle in each minute was then manually calculated and used as a representative diaphragm activation of each minute of the cycling test (Figure 3). In a similar way, IC maneuvers were manually identified and activation data retrieved. In summary, while the method involves some manual steps it is not possible to manually manipulate EMGdi amplitude signals within separate breaths. The method is therefore (in contrast to the manual method) not prone to inter rater variability. Differences in outcomes could only occur in case of not selecting appropriate minute intervals or IC maneuvers.”

The reported results used for analysis were taken from one of the five channels that (on average) contained the largest EMGdi signals during IC maneuvers.

### Exercise Testing

All patients underwent constant work rate (CWR) cycling tests consisting of 3 min of resting, 1 min of unloaded cycling and immediately followed by cycling against 75% of the patient’s peak work rate achieved during a maximal incremental cardiopulmonary exercise test (CPET) (American Thoracic Society/American College of Chest Physicians [ATS/ACCP], 2003) until symptom limitation. The tests were conducted on an electrically braked cycle ergometer (Ergometrics 900, Ergoline, Blitz, Germany) with detailed metabolic (SensorMedics Vs229d, Acertys Healthcare, Aartselaar, Belgium) and cardiopulmonary measurements (Cardiosoft, Acertys Healthcare, Aartselaar, Belgium). The respiratory flow signal was recorded during the exercise to be able to identify the respiratory cycle. ECG recordings were obtained via an impedance cardiography device (PhysioFlow, Manatec Biomedical, Folschviller, France) validated for COPD patients and recorded as a separate channel (Louvaris et al., 2019). Analog outputs of all variables (i.e., respiratory flow, five EMGdi and ECG) were collected in separate channels with a data acquisition unit (Micro1401-3, Cambridge Electronic Design Limited, Cambridge, United Kingdom). Data channels were synchronously collected by the same system and processed with the same acquisition software (Spike 2, Cambridge Electronic Design Limited, Cambridge, United Kingdom). The system was *a priori* checked for potential time delays between the different systems providing the signals of the different channels (e.g., ECG and EMGdi). No time delays were present and therefore, no additional post collection synchronization of data had to be performed.

### Inspiratory Muscle Training (IMT)

Inspiratory muscle training was performed daily by four subjects using an electronic POWERbreathe^®^ KH2 device (HaB International Ltd., Southam, United Kingdom) for 8 weeks, according to a previously published protocol (Langer et al., 2015). In short, the patients trained at the highest tolerable intensity, 30 breaths per session and two sessions per day. Progression of training intensity and MIP measurements were performed weekly.

### Statistical Analyses

Comparisons of diaphragm activation at pre-measurement and pre/post IMT differences obtained from two assessors using the manual signal processing method and with the semi-automated signal processing method were made. Pearson’s correlation coefficient (r) was used to establish associations between measurements. The intra-class correlation coefficient (ICC) based on a mean-rating, absolute-agreement, 2-way mixed-effects model was used to quantify agreement between two assessors (inter-rater reliability) and between the two methods. Agreement of the results from two assessors and between the two methods was assessed by plotting mean differences between assessors or methods against average values (Bland-Altman plots) (Bland and Altman, 1986). Limits of agreement were defined as ±1.96 × standard deviation of the difference between the two methods, corresponding to 95% confidence intervals (CI). The interaction over time between the two assessors and the two methods was assessed using repeated measures ANOVA. Within rater Coefficient of Variation (CV) for the two raters was calculated from five representative breaths during resting and at the end of exercise. For the semi-automated method, the CV was also calculated at the same time points. Statistical analyses were performed using GraphPad Prism version 8 for Windows (GraphPad Software, La Jolla, CA, United States) and IBM SPSS Statistics 25.0 Desktop (IBM Corp., Armonk, NY, United States). Statistical significance was considered at *p* < 0.05. Data are presented as means ± SD.

## Results

Datasets supporting the conclusions of this manuscript are available on request. Characteristics of included patients are presented in Table 1. Patients exhibited moderate to severe airway obstruction with static hyperinflation, reduced exercise capacity, and inspiratory muscle strength. There are approximately four heartbeats during one inspiratory cycle both at rest and during exercise (Table 1).

**TABLE 1:** Patient characteristics: pulmonary function, respiratory muscle strength, maximal, and endurance exercise capacity.

	**All subjects (*n* = 7)**
**General characteristic**	
Male:female	4:3
Age, years	66 ± 5
BMI, kg/m^2^	25 ± 7
**Pulmonary function**	
FEV_1_, L (%pred)	1.37 ± 0.57 (56 ± 31)
FEV_1_/FVC, %	42 ± 15
IC, L (%pred)	1.96 ± 0.44 (76 ± 27)
FRC, L (%pred)	5.14 ± 1.85 (161 ± 42)
RV, L (%pred)	3.64 ± 1.48 (149 ± 57)
TLC, L (%pred)	7.10 ± 1.73 (122 ± 19)
D_L_CO, mmol/min/Kpa (%pred)	4.63 ± 2.01 (59 ± 25)
**Respiratory muscle strength**	
MIP at RV, cmH_2_O (%pred)	−77 ± 11 (85 ± 18)
Pdimax, cmH_2_O	89 ± 19
MEP at TLC, cmH_2_O (%pred)	167 ± 55 (99 ± 34)
**Symptom-limited peak incremental cycling ergometer exercise test**	
Cycling duration (minutes)	7.7 ± 1.5
Peak work rate, W (%pred)	82 ± 27 (64 ± 24)
VO_2_, L/min (%pred)	1.40 ± 0.60 (74 ± 33)
HR, bpm (%pred HRmax)	118 ± 16 (76 ± 9)
Ventilation, L/min (%MVV)	44.6 ± 7.6 (88 ± 13)
**Constant work rate cycling test (CWR cycling)**	
Cycling work rate, W (%Wmax)	59 ± 20 (72 ± 3)
Cycling duration, min	8.0 ± 3.7
Resting HR, bpm (%pred HRmax)	80 ± 10 (52 ± 16)
HR at end exercise, bpm (%pred HRmax)	124 ± 17 (80 ± 10)
Resting BF, bpm	22 ± 8
BF at end exercise, bpm	32 ± 8
HR:BF at rest, per min	3.9 ± 1.1
HR:BF at end exercise, per min	4.1 ± 1.2
Ventilation, L/min (%MVV)	38.3 ± 8.4 (80 ± 11)
Resting Ti, s	1.1 ± 0.5
Ti at end exercise, s	0.8 ± 0.2
Resting Te, s	2.1 ± 0.7
% change from rest Ti	−25 ± 22
% change from rest Te	−36 ± 23

*FEV_1_, forced expiratory volume in one second; FVC, forced vital capacity; IC, inspiratory capacity; FRC, functional residual capacity; RV, residual volume; TLC, total lung capacity; D_*L*_CO, diffusing capacity of the lung for carbon monoxide; MIP at RV, maximal inspiratory mouth pressure at residual volume; PdiMax, maximal transdiaphragmatic pressure during sniff maneuver; MEP at TLC, maximal expiratory mouth pressure at total lung capacity; VO_2_, oxygen consumption; HR, heart rate; MVV, maximal voluntary ventilation; BF, breathing frequency; Ti, inspiratory time; Te, expiratory time; %pred, %predicted.*

### Comparisons of EMGdi/EMGdiMax% Obtained by Either the Two Assessors or as Processed With the Semi-Automated Method From Data Collected During a Constant Work Rate Cycling Task

**FIGURE 4:**
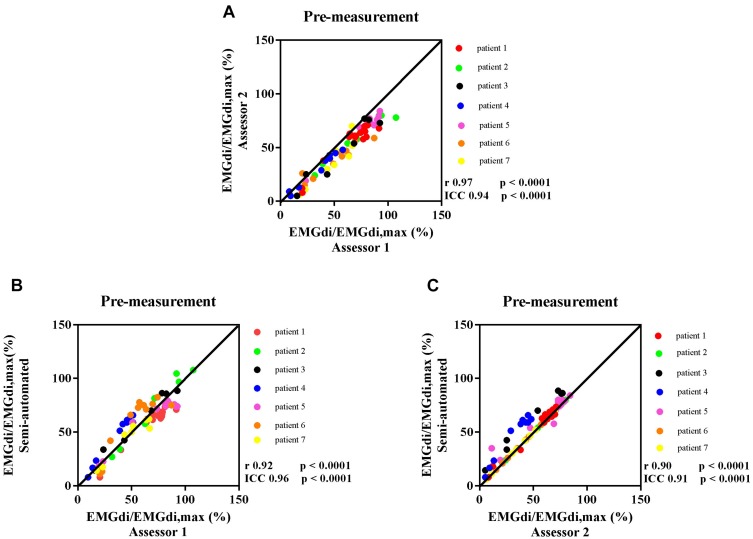
The correlation between EMGdi/EMGdiMax% calculated from the manual method by two assessors during the pre-measurement CWR cycling test **(A)**; the semi-automated method and the manual method by assessor 1 **(B)**; and the semi-automated method and the manual method by assessor 2 **(C)**. Line of identity, linear regression coefficients, intra-class correlation coefficients (ICC), and significances are presented in each figure. Each of the data points represents the activation of diaphragm EMG (EMGdi/EMGdiMax%) of each patient in every minute during the pre-measurement CWR cycling test.

The intra-class correlation coefficients (ICC) between diaphragm activation signals obtained with the manual methods by two assessors at pre-measurement was 0.94, *p* < 0.0001, 95% CI: 0.17–0.98 (Figure 4A). The ICC between EMGdi signals from the semi-automated method and the results obtained by using the manual method from assessor 1 and 2 at pre-measurement were 0.96, *p* < 0.0001, 95% CI: 0.94–0.97 (Figure 4B) and 0.91, *p* < 0.0001, 95% CI: 0.60–0.97 (Figure 4C), respectively.

**FIGURE 5:**
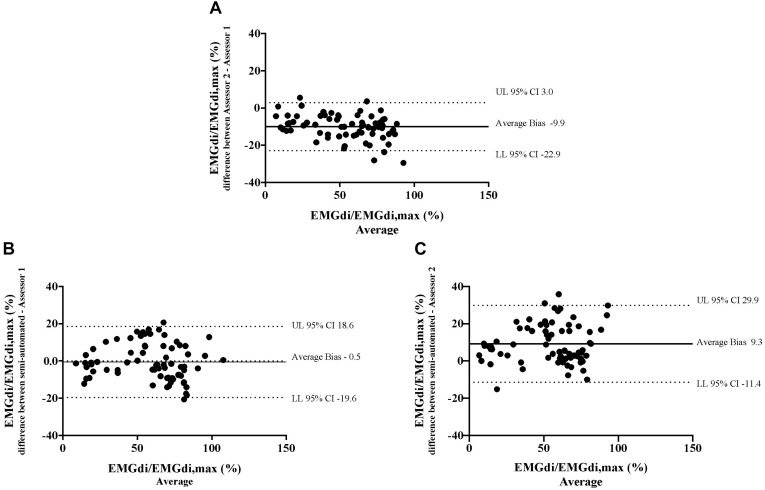
The results from the manual method by two assessors and the semi-automated method are compared in seven patients in each minute during the pre-measurement CWR cycling test. Bland-Altman plots of EMGdi/EMGdiMax% calculated from the manual method by two assessors **(A)**; the semi-automated method and the manual method by assessor 1 **(B)**; the semi-automated method and the manual method by assessor 2 **(C)**.

Bland – Altman plots for the agreement of EMGdi/EMGdiMax% for the above-mentioned comparisons are presented in Figures 5A–C. On average, the EMGdi/EMGdiMax% obtained from the manual method by assessor 2 resulted in lower values than those obtained from assessor 1 (average bias of the differences: −9.9%; CI: −22.9 to 3.0%, Figure 5A). The plot of agreement between EMGdi/EMGdiMax% values from the semi-automated method and results of the manual method obtained by assessor 1 showed that on average the values from the semi-automated method were very similar with the values obtained from the manual method by assessor 1 (average bias of the differences: −0.5%; CI: −19.6 to 18.6%, Figure 5B). The plot of agreement between the values from the semi-automated method and those obtained by the manual method from assessor 2 showed higher values from the semi-automated method (average bias of the differences: 9.3%; CI: −11.4 to 29.9% Figure 5C).

**FIGURE 6:**
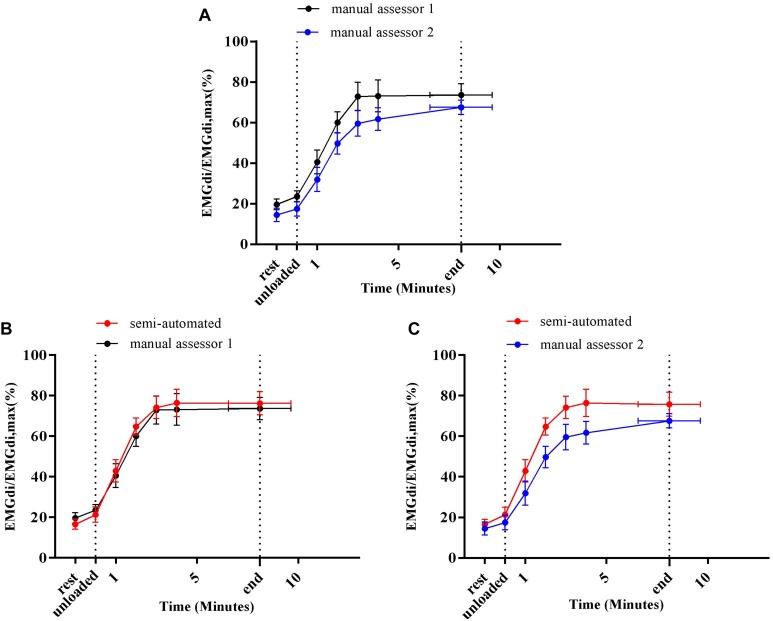
Average diaphragm activation (EMGdi/EMGdiMax%) of seven patients during CWR cycling test at pre-measurement calculated from the manual method by two assessors **(A)**; the semi-automated method and the manual method by assessor 1 **(B)**; the semi-automated method and the manual method by assessor 2 **(C)**.

Average EMGdi/EMGdiMax% obtained from the manual method by two assessors and values obtained with the semi-automated method were plotted against time for each minute during CWR cycling are presented in Figures 6A–C. There were no significant method by time interactions observed neither between the values from two assessors (Figure 6A; *P* = 0.24), nor between values from the semi-automated method and assessor 1 (Figure 6B; *P* = 0.30), or the semi-automated method and assessor 2 (Figure 6C; *P* = 0.11).

Average absolute maximal activation values (obtained during IC maneuvers) obtained by assessor 1 and 2 with the manual analysis method of were 0.146 ± 0.062 volt and 0.150 ± 0.060 volt, respectively. No significant differences were found between the maximal activation values obtained by assessor 1 and 2 (*P* = 0.25).

The CV of assessor 1 was 22% at rest and 13% at the end of exercise at pre-measurement. At post-measurement, the CV was 21% at rest and 26% at end exercise. For assessor 2 the CV was 54% at rest and 11% at the end of exercise. At post-measurement, the CV was 28 and 20% at rest and end exercise, respectively. The CV calculated from the semi-automated were 13 and 11% at rest and end exercise, respectively, at pre-measurement. At post-measurement, the CV was 10 and 12% at rest and end exercise, respectively.

### Comparisons of the Pre/Post Intervention Differences in EMGdi/EMGdiMax% Obtained by Either the Two Assessors or as Processed With the Semi-Automated Method From Data Collected During a Constant Work Rate Cycling Task

**FIGURE 7:**
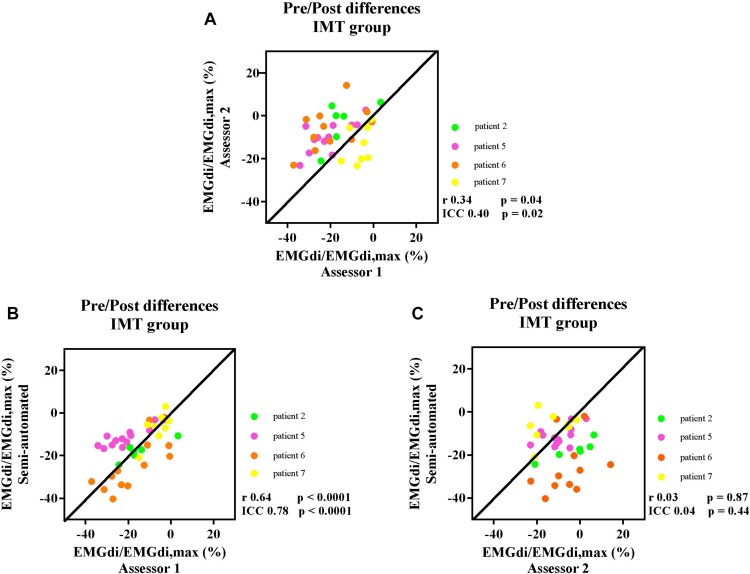
The correlation between the pre/post difference in four participants EMGdi/EMGdiMax% calculated from the manual method by two assessors **(A)**; the semi-automated method and the manual method by assessor 1 **(B)**; the semi-automated method and the manual method by assessor 2 **(C)**. Line of identity, linear regression coefficients and intra-class correlation coefficients (ICC) and significances are presented in each figure. Each of the data points represents the pre/post differences of diaphragm EMG activation (EMGdi/EMGdiMax%) pre and post the intervention of each patient in every minute during CWR cycling test.

After 8 weeks of IMT, inspiratory muscle function was improved in four patients that had completed the IMT protocol [two men and two women, age 64 ± 4 years, BMI 25 ± 7 kg/m^2^, FEV_1_ 1.56 ± 0.69 L (63 ± 41 %predicted)] who participated in the IMT intervention. Maximal inspiratory pressure (MIP) improved from −77 ± 15 cmH_2_O (84 ± 16 %predicted) to −91 ± 25 cmH_2_O (100 ± 30 %predicted). Maximal transdiaphragmatic pressure (Pdi) measured during maximal inspiratory sniff maneuvers improved from 93 ± 21 cmH_2_O to 105 ± 24 cmH_2_O. The average cycling duration was 8.4 ± 2.5 min at pre-measurement and 16.4 ± 7.8 min at post-measurement. The pre/post IMT differences in EMGdi/EMGdiMax% during cycling before and after IMT, were calculated. The correlations between the pre/post IMT differences in EMGdi/EMGdiMax% calculated from the manual method by two assessors and the semi-automated method during the CWR cycling test before and after the intervention are presented in Figures 7A–C.

The ICC between the values of pre/post differences from assessor 1 and assessor 2 was 0.40, *P* = 0.02, 95% CI: −0.09 to 0.68 (Figure 7A). The ICC between the pre/post IMT differences from the semi-automated method and assessor 1 was 0.78, *p* < 0.0001, 95% CI: 0.58–0.89 (Figure 7B), while the ICC between the pre/post IMT differences from the semi-automated method assessor 2 was 0.04, *P* = 0.44, 95% CI: −0.58 to 0.46 (Figure 7C).

**FIGURE 8:**
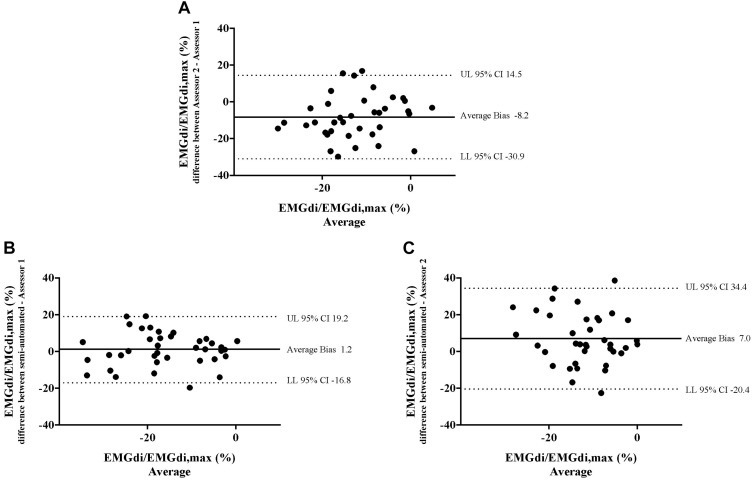
The results from the manual method by two assessors and the semi-automated method are compared in four patients in each minute during CWR cycling test pre and post the intervention. Bland-Altman plots of the pre/post differences in EMGdi/EMGdiMax% calculated from the manual method by two assessors **(A)**; the semi-automated method and the manual method by assessor 1 **(B)**; the semi-automated method and the manual method by assessor 2 **(C)**.

Bland – Altman plots for the agreements of pre/post IMT differences in EMGdi/EMGdiMax% calculated from two analyzing methods are presented in Figures 8A–C. On average, the pre/post IMT differences in EMGdi/EMGdiMax% obtained from the manual method from assessor 2 was lower than assessor 1 (average bias of differences: −8.2%; CI: −30.9 to 14.5%, Figure 8A). The pre/post IMT differences in EMGdi/EMGdiMax% obtained from the semi-automated method was on average similar to values obtained with the manual method by assessor 1 (average bias of differences: 1.2%; −16.8 to 19.2%, Figure 8B). The pre/post differences values from the semi-automated method are higher than the values from the manual method by assessor 2 (average bias of differences: 7.0%; CI: −20.4 to 34.4%, Figure 8C).

**FIGURE 9:**
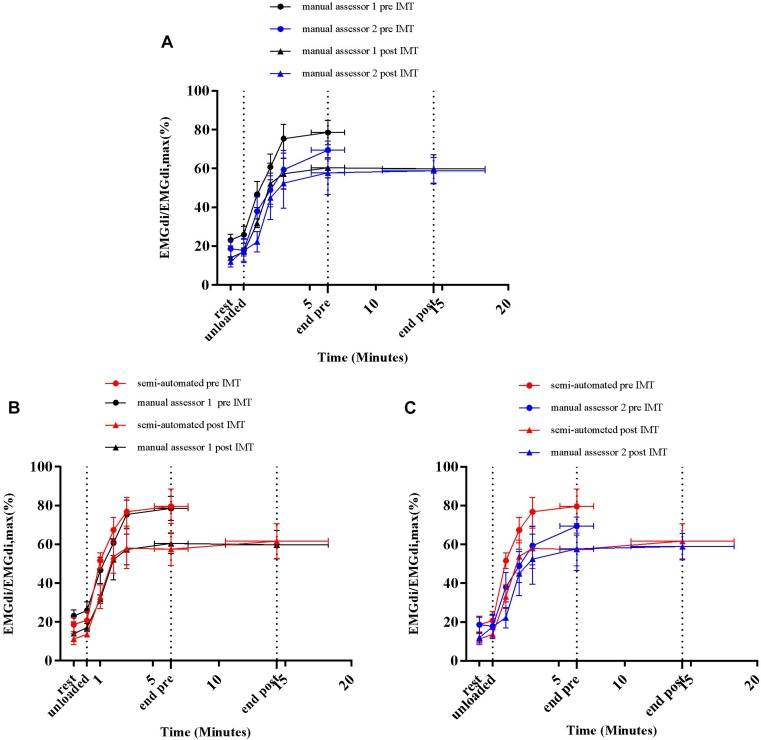
Average diaphragm activation (EMGdi/EMGdiMax%) of four patients during CWR cycling test pre and post the intervention calculated from the manual method by two assessors **(A)**; the semi-automated method and the manual method by assessor 1 **(B)**; the semi-automated method and the manual method by assessor 2 **(C)**.

Average EMGdi/EMGdiMax% values obtained from the manual method by the two assessors and the semi-automated method were plotted against time for each minute during CWR cycling performed pre and post the IMT intervention period (Figures 9A–C). There were no significant method by time interactions observed between the values from two assessors (Figure 9A; *P* = 0.29), the semi-automated method and assessor 1 (Figure 9B; *P* = 0.55) and the semi-automated method and assessor 2 (Figure 9C; *P* = 0.50).

Average absolute maximal activation values (during IC maneuver) obtained with the manual analysis method by assessor 1 and 2 were 0.121 ± 0.075 volt and 0.124 ± 0.072 volt, respectively, at pre-measurement, and 0.158 ± 0.101 volt and 0.142 ± 0.92 volt, respectively, at post-measurement (*P* = 0.62 and *P* = 0.20, respectively).

### Duration of Analysis Between Manual and Semi-Automated Method

The average duration of 11 CWR cycling tests (seven at pre-measurement and four at post measurement), including resting and unloaded cycling, was 13 ± 6 min (range 4.2 to 22.0 min). The analyzing time using the manual method was 82 ± 20 min (range 63 to 115 min) for assessor 1 and for the semi-automated method was 29 ± 9 min (range 18–49 min). Difference between methods 53 ± 15 min (*p* < 0.0001).

## Discussion

### Main Findings

We validated a custom developed ECG removal method for EMG amplitude analysis against a commonly used manual approach. The main findings of this study are that the newly developed semi-automated EMGdi analysis method is more time efficient and that it will be less prone to the inter-rater variability that was observed when the manual method was applied by two independent assessors. EMGdi amplitudes obtained with the semi-automated method agreed well with values obtained by one of the two manual assessors. The findings suggest that EMGdi analysis using the proposed semi-automated method can be used to evaluate changes in EMG amplitudes over a wide range of minute ventilations recorded at rest and during exercise in patients with COPD.

### Inter-Rater Reliability of the Manual Method

Resting diaphragm activation (EMGdi/EMGdiMax%) values obtained by both assessors using the manual method ranged from 10 to 20% (Figures 6A,B, 9A,B). During the CWR cycling exercise, this activation increased steeply at the beginning of the exercise and reached a plateau until the exercise was terminated by patients’ symptom limitation (Figures 6A,B, 9A,B). Similar patterns were observed in previous studies (Luo and Moxham, 2005; Luo et al., 2011; Langer et al., 2018). The observed differences of 8–9% in EMGdi amplitudes between raters are, however, substantial and might impact on the ability to detect differences in EMG amplitudes after interventions (Figures 5A, 8A). Along these lines the pre/post IMT differences manually obtained by assessor 1 resulted in a reduction of approximately 20% of EMGdi/EMGdiMax at iso-time (Figures 9A,B), whereas analyses performed by assessor 2 resulted in a much smaller reduction of approximately only 10% (Figures 9A,C). In an attempt to explain these differences we looked into the manual analyses as performed by the two assessors in more detail.

**FIGURE 10:**
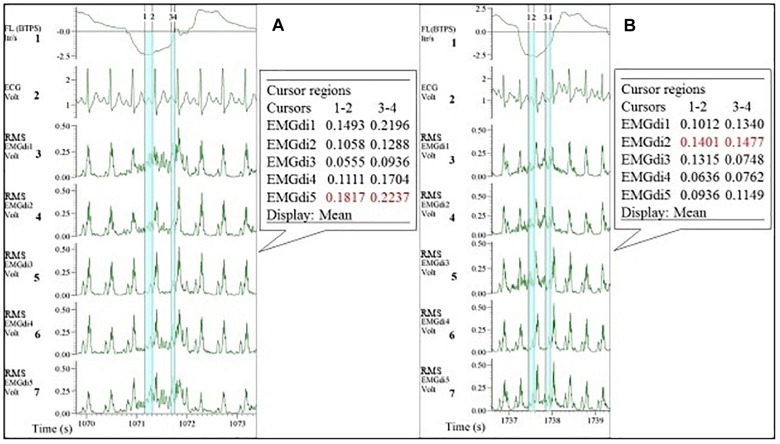
Example of EMGdi recordings during cycling exercise toward the end of the (symptom limited) cycling test at the pre- **(A)** and post- **(B)** measurement for the analysis using the manual method. Channel 1: respiratory flow (l/s; negative flow indicating the inspiratory cycle), Channel 2: ECG recording (volt), Channels 3–7: diaphragm EMG (RMS EMGdi) recordings. The periods highlighted in light blue are the possible periods that could be chosen without ECG contamination in an inspiratory cycle. Numbers in cursors regions boxes show the mean EMGdi values between two vertical cursors. The numbers in red indicate that the differences in the mean values between cursors 1–2 are closer to the values between cursor 3–4 at post-measurement.

Since EMGdiMax (volt) values obtained by the two assessors were similar, the differences in the EMGdi/EMGdiMax ratio between the two assessors must have originated from the selection of the EMGdi signal between QRS complexes of the tidal breaths. Retrospectively, we observed that in most cases there were several intervals between QRS complexes that assessors could select for their analyses (Figure 2). Upon closer inspection we further realized that assessor 1 systematically tended to choose the period that resulted in the “highest” EMGdi value of each inspiratory cycle (frequently occurring toward the very end of an inspiratory cycle), while assessor 2 always chose intervals that contained the “widest” available signal typically located more “centrally” within each inspiratory phase. This is illustrated in Figure 2. While assessor 1 systematically selected the period between cursor 3 to 4, assessor 2 tended to choose the period between cursor 1–2. We noticed that especially during pre-IMT assessments the amplitude of EMGdi was higher toward the end of each inspiratory cycle, indicating more pronounced diaphragm activation toward the end of the inspiratory cycle (Figure 10A). Since the given illustrative example occurred frequently during the tests the intervals selected by assessor 1 often resulted in higher values than the intervals chosen by assessor 2 (Figures 5A, 8A).

**FIGURE 11:**
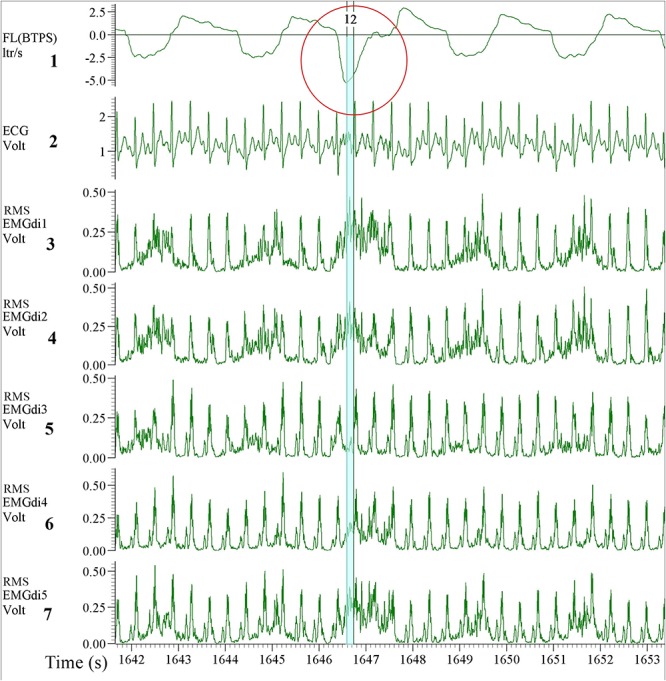
Example of EMGdi recordings during a cycling exercise toward the end of the (symptom limited) cycling test for the analysis using the manual method. Channel 1: respiratory flow (l/s; negative flow indicating the inspiratory cycle), Channel 2: ECG recording (volt), Channels 3–7: Diaphragm EMG (RMS EMGdi) recordings. The IC maneuver is highlighted in the red circle indicated by the higher flow, which accompanies the maximal activation of the EMGdi. The periods highlighted in light blue are the periods that could be chosen without ECG contamination in an inspiratory cycle during IC maneuver. With the short inspiratory time during the IC maneuver, it left only one available (light blue) period that EMGdi could be retrieved.

As stated earlier the values of EMGdiMax were not significantly different between two assessors. This initially seems surprising given the different approaches taken by the two assessors as described above. It can be explained, however, based on the shorter inspiratory period (Ti) during the IC maneuvers (during which the EMGdiMax signals were obtained) in comparison to the tidal breaths (during which the EMGdi intervals were selected). As illustrated in Figure 11 during the short inspiratory periods of the IC maneuvers there was frequently only a single EMG interval between QRS complexes available to select. This can most likely explain the smaller differences in EMGdiMax values between assessors in comparison to EMGdi.

Interestingly there was also less disagreement between the results from assessor 1 and 2 during the post-intervention analyses in comparison to pre-intervention comparisons (Figure 9A). Despite both assessors treating the EMGdi data with the same approach as for the pre-intervention measurements (i.e., one rater looking for the highest while the other looked for the widest available signal interval), the values from assessor 2 were closer to the values from assessor 1. This finding can probably be explained by previously reported pre/post differences in EMG amplitude signal patterns over time, during muscular activation, in response to muscle training (Oliveira Ade and Goncalves, 2009). Known effects of muscle training, including inspiratory muscle training, are improvements in force output and motor learning, thereby decreasing muscle activation levels at iso-loads (Campos et al., 2002; Lay et al., 2002). In fact, higher EMGdiMax and decreased relative activation of the diaphragm (i.e., lower muscle activity) after training at iso-loads were previously reported by our group (Langer et al., 2018).

As shown in Figure 10A and as mentioned earlier, during pre-IMT assessments, the EMGdi signal from the diaphragm increased from the beginning toward the end of the inspiration. The EMGdi values (volt) between cursors 1–2 were always lower than those between cursors 3–4 (Figure 10A). The numbers marked in red are the values taken as a representative mean EMGdi of that breath (Figures 10A,B). After inspiratory muscle training, however, EMGdi values earlier during inspiration were less different from those toward the end of the inspiratory phase (Figure 10B). It therefore seems like patterns that had previously been observed after resistance training of peripheral muscles (reduced EMG/time slopes after training) (Oliveira Ade and Goncalves, 2009), were also detected in our diaphragm EMG data. These findings can probably help to explain why inter-rater differences, despite of using similar approaches, were less pronounced after the resistance training period.

### Comparisons Between Manual and Semi-Automated EMGdi Analyses

Data from the manual analyses of assessor 1 (who searched for the intervals containing the EMGdi signal with highest amplitude for every breath) resulted in good agreement with the results from the semi-automated method. We assume that the semi-automatically processed data are most representative of the “real” EMGdi values since the ECG contamination was eliminated from the signal. Based on these findings the analysis strategy of assessor 1 should probably be favored (i.e., selecting the EMGdi interval in between QRS complexes that provides the “highest” amplitude) should probably be favored above the approach taken by assessor 2. This is further supported by the fact that magnitude of pre-post intervention differences of both assessor 1 and from the semi-automated method are in line with findings from a previous study that assessed diaphragm activation during the CWR cycling test before and after a similar IMT intervention (Langer et al., 2018).Therefore, if the manual method should be used, we would recommend to manually select EMG parts between QRS complexes that result in the “highest” average EMGdi (i.e., selecting intervals toward the end of each inspiratory period). This strategy of manual analysis showed a good agreement with the semi-automated method on a group level, suggesting that both methods can be used interchangeably. The discussion onward will focus on the comparisons between the results of the semi-automated method and values obtained with the manual method from assessor 1.

Bland – Altman plots of both pre-intervention measurements and pre/post IMT differences in EMGdi/EMGdiMax% showed good overall agreement. Considering the differences, which scattered randomly above and below zero, it did not appear as if there were systematic over- or underestimations present, or that differences between methods became larger when activation was higher (i.e., at higher minute ventilation during cycling) (Figures 5B, 8B).

**FIGURE 12:**
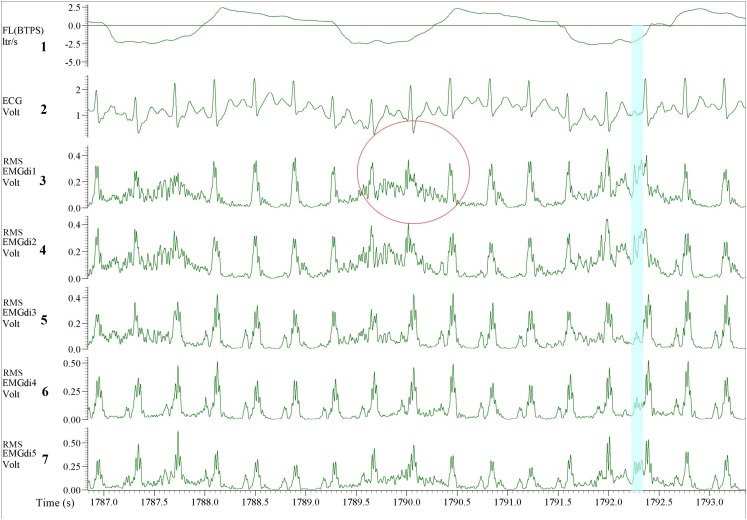
Illustration of the EMGdi recordings during cycling exercise toward the end of the (symptom limited) cycling test when the inspiratory time (Ti) is shorter. Channel 1: respiratory flow (l/s; negative flow indicating the inspiratory cycle), Channel 2: ECG recording (volt), Channel 3–7: diaphragm EMG (RMS EMGdi) recordings. Several QRS complexes appear during the inspiratory cycle which is already a short period. The QRS complexes take up a large part during the inspiratory cycle resulting in less “clean” EMGdi to be selected. The red circle shows one inspiratory cycle when the QRS complex appears precisely at the peak of the amplitude of the EMGdi. The EMGdi buried under this QRS complex cannot be retrieved. Therefore, the assessor must choose the part outside of the QRS complex, which results in lower mean EMGdi value, thus underestimates the diaphragm activation. The blue highlight shows a small period of peak EMGdi. The mean EMGdi value of this period will be higher (average of high values in the short time) therefore the EMGdi being overestimated.

Two factors probably contribute to the relatively wide (±20%) limits of agreement that were observed between methods. Firstly, during manual analyses QRS complexes occurring during the inspiratory cycles can cover major parts of the inspiratory EMG signal. These parts (which might contain the highest activation portion during a given inspiration) are consequently not available for analysis (Figure 12). This limited availability of EMGdi signal probably contributed to either over- or underestimation of the manual signal in contrast to the semi-automatically processed signal which could always take the full inspiratory period (i.e., from zero flow to zero flow) into consideration.

A second factor that probably contributes to the width of the limits of agreement between methods is the fact that the average EMGdi from the manual method is obtained from a representative sample of five consecutive breaths toward the end of each exercise minute. In contrast during the semi-automated method, all breaths performed during each minute are analyzed (Luo and Moxham, 2005). It might very well be that the sample of five breaths is not always a perfect representation of average EMGdi during a given minute of breathing resulting in between method differences on a minute-by-minute basis. The overall agreement of the EMGdi/EMGdiMax between two methods on a group level was, however, good, and no significant method by time interaction effects were observed (Figures 6B, 9B). This suggests that both methods can be used interchangeably on a group level.

### Degree of Variation

The CV were calculated from the manual analyses performed by two raters were mostly higher than the CV calculated from the semi-automated method. This lower degree of variability when using the semi-automated method will probably increase the ability to detect true differences between measurement conditions. The reduction in variability is most likely due to both the absence of noise within breaths as well as the fact that instead of a representative sample of five breaths all breaths of each minute were used for analyses.

### Clinical Implications

The EMGdi/EMGdiMax ratio is currently being used as a surrogate of neural respiratory drive (NRD) and both magnitude as well as changes in NRD have been shown to be closely related to (changes in) dyspnea sensation (Jolley et al., 2015; Langer et al., 2018) which is an important symptom in patients with chronic obstructive and restrictive lung diseases (Parshall et al., 2012). It is essential to obtain correct values of diaphragm activation to be able to interpret the results linked with the patient’s symptoms and also to reliably detect changes induced by different interventions. A reliable and objective method to process these data is beneficial for breathlessness management in patients with COPD both in research and clinical routine.

### Strengths and Limitations

The semi-automated method was designed to overcome several shortcomings of the manual method. By automatically removing ECG artifacts throughout the recording, the analysis time is shortened by more than half. After the ECG artifact was removed the resulting “clean” EMGdi signal could be integrated over the full inspiratory cycle. This integration of EMG activity over the course of a contraction is a common practice for other skeletal muscles but was not possible with the manual EMGdi analysis methods available so far. In addition it facilitates the performance of breath-by-breath analyses which allows all data points to be considered. This is the first time that the inter-rater reliability of the often used “manual method” has been evaluated. We were for the first time able to identify several sources for inter-rater variability which should be eliminated by the more objective, semi-automated processing of full inspirations that have previously been cleaned of ECG artifacts by our newly developed method.

Limitations of our study are the relatively small sample size and the absence of an age-matched control group. The results would need to be confirmed in a larger sample of subjects performing different types of exercise tests resulting in a large variability of heart rate and ventilation responses. In addition, inclusion of a healthy age-matched control group and comparisons of findings with COPD patients would have allowed further investigations into the validity of the semi-automated method at rest and during exercise. Since our methods were only compared in a specific group of patients future studies might be required to further validate the use of the semi-automated EMGdi analysis in other populations. In our study EMGdi signals were evaluated only at the extremes of activation (i.e., resting breathing and close to maximal activation). The model/approach has not been tested over a range of intensities (and as such diaphragm activity). It would have been preferable to evaluate the responses over a broader range of minute ventilations and heart rate (e.g., during a stepwise maximal incremental exercise test). Further study is also needed with regard to responsiveness of the signals and reproducibility of findings both before and after different pharmacological and non-pharmacological interventions that are supposed to reduce respiratory effort and symptoms of breathlessness.

## Conclusion

The semi-automated ECG artifact removal method for EMGdi analyses will be helpful to eliminate sources of inter-rater variability that were observed between different raters applying the manual method. Therefore the semi-automated method offers a more objective approach for analyzing EMGdi data while at the same time requiring significantly less analyzing time. We propose this method as a new standard for objective EMGdi amplitude analyses in the future.

## Data Availability

The datasets generated for this study are available on request to the corresponding author.

## Ethics Statement

The Ethical Committee Research of KU Leuven/UZ Leuven, Belgium approved the study (S58513).

## Author Contributions

SD, RG, and DL contributed to the conception and design of the study. SD, ZL, and LoJ contributed to the acquisition. LuJ developed the analysis algorithm. SD and AR performed the data analysis. SD organized the database, performed the statistical analysis, and wrote the first draft of the manuscript. LuJ, AR, RG, and DL wrote sections of the manuscript. All authors contributed to the manuscript revision, and read and approved the submitted version.

## Conflict of Interest Statement

The authors declare that the research was conducted in the absence of any commercial or financial relationships that could be construed as a potential conflict of interest.
